# Dr. Voelcker's Address on Regeneration

**Published:** 1918-10-19

**Authors:** 


### Dr. Voelcker's Address on Regeneration.

At the opening meeting of the Medical Society of London on Monday, October 14, the address of the new president, Major A. F. Voelcker, M.D. Lond., F.R.C.P., was devoted to the subject of Regeneration.

He said reconstruction was now in the air, and the medical, like the other professions, would be striving to bring about a new era. The most optimistic medical man could not be satisfied with the position which the profession occupied at the present time; the services of the medical man were not even now understood, and therefore they were not appreciated. In the regenerated profession he urged the Fellows of this society to take their part as leaders, not as appendages in the various fields of activity concerned with the health and welfare of the community.

With regard to the sending of medical men to Parliament, could it be said that a fifth, a medical, party in Parliament would add to its utility? He thought that the less medical men had to do with professional politics the better. The Health Minister should be the chosen of the profession, not of any political party. The creation of such a Ministry would not, however, absolve the doctor from his daily campaign against indulgence and ignorance.

He thought there could be no doubt that the public was obsessed with the glamour of specialism. In the future better days he hoped that the practice of specialists would flow to them through the channel of the practitioner, not through the press or the public. And it would be one of the dangers of a State-controlled medicine that the popular clamour for specialism might lead to the multiplication of special hospitals or special departments. For years the managing bodies of our hospitals had had to realise that some change must take place in the financial resources of the hospitals for the continued carrying on of efficient work. Some felt it would be an evil day when the State, by its Medical Service, undertook the care of the health of the individual.

He concluded by a sympathetic word for nurses. He hoped that in a regenerated state of the medical profession doctors would see to it that the remuneration of those whose work was so essential to the success of treatment, and whose labours were so splendidly and so ungrudgingly given—the nurses—would no longer be on a scale which was a scandal to the profession. In his view a woman who took up nursing took ten years off her life. After training three or four years she was able to earn a wage which a parlourmaid would scorn. The only attraction he could see was that in it a woman was able to carry out her ideals of life and conduct.

